# SVM classifier to predict genes important for self-renewal and pluripotency of mouse embryonic stem cells

**DOI:** 10.1186/1752-0509-4-173

**Published:** 2010-12-21

**Authors:** Huilei Xu, Ihor R Lemischka, Avi Ma'ayan

**Affiliations:** 1 Department of Pharmacology and System Therapeutics, Mount Sinai School of Medicine, 1 Gustave L. Levy Place, New York, New York, 10029, USA; 2 Systems Biology Center New York (SBCNY), Mount Sinai School of Medicine, 1 Gustave L. Levy Place, New York, New York, 10029, USA; 3 Department of Gene and Cell Medicine, Mount Sinai School of Medicine, 1 Gustave L. Levy Place, New York, New York, 10029, USA; 4 Black Family Stem Cell Institute, Mount Sinai School of Medicine, 1 Gustave L. Levy Place, New York, New York, 10029, USA

## Abstract

**Background:**

Mouse embryonic stem cells (mESCs) are derived from the inner cell mass of a developing blastocyst and can be cultured indefinitely in-vitro. Their distinct features are their ability to self-renew and to differentiate to all adult cell types. Genes that maintain mESCs self-renewal and pluripotency identity are of interest to stem cell biologists. Although significant steps have been made toward the identification and characterization of such genes, the list is still incomplete and controversial. For example, the overlap among candidate self-renewal and pluripotency genes across different RNAi screens is surprisingly small. Meanwhile, machine learning approaches have been used to analyze multi-dimensional experimental data and integrate results from many studies, yet they have not been applied to specifically tackle the task of predicting and classifying self-renewal and pluripotency gene membership.

**Results:**

For this study we developed a classifier, a supervised machine learning framework for predicting self-renewal and pluripotency mESCs stemness membership genes (MSMG) using support vector machines (SVM). The data used to train the classifier was derived from mESCs-related studies using mRNA microarrays, measuring gene expression in various stages of early differentiation, as well as ChIP-seq studies applied to mESCs profiling genome-wide binding of key transcription factors, such as Nanog, Oct4, and Sox2, to the regulatory regions of other genes. Comparison to other classification methods using the leave-one-out cross-validation method was employed to evaluate the accuracy and generality of the classification. Finally, two sets of candidate genes from genome-wide RNA interference screens are used to test the generality and potential application of the classifier.

**Conclusions:**

Our results reveal that an SVM approach can be useful for prioritizing genes for functional validation experiments and complement the analyses of high-throughput profiling experimental data in stem cell research.

## Background

Mouse embryonic stem cells (mESCs) are derived from the inner cell mass of a developing blastocyst and can be cultured indefinitely in-vitro. Their distinct features are their ability to self-renewal as well as to differentiate into all adult cell types including the germ-line. These features render mESCs ideal for applications in basic scientific research and translational medicine. To harness their full potential, better understanding of the molecular mechanisms of mESCs self-renewal maintenance and pluripotency is critical. Therefore, genes that are critical to mESCs self-renewal maintenance are of interest to the stem cell research field. In the past decade, significant steps have been made toward identifying and characterizing the genes and regulatory networks that compose the self-renewal machinery. A mESCs stemness membership gene (MSMG) signature has been proposed through application of high-throughput profiling approaches such as mRNA expression microarrays combined with advanced computational analyses as well as through low-throughput detailed functional studies [1-3]. Genes that are predominantly expressed in mESCs cells are considered putative candidates for being MSMGs. Nevertheless, the overlap among candidate MSMGs across different studies is surprisingly small, whereas the full identification of MSMGs, the genes responsible for self-renewal and pluripotency, remains largely incomplete.

Fuelled by the growing volume, diversity and complexity of genome-wide profiling data generated from high-throughput biotechnologies, advanced computational approaches such as machine learning have been used to analyze multi-dimensional experimental data and integrate results from many studies [4-10]. Support Vector Machines (SVM) is a popular supervised machine learning method that is based on statistical learning theory [11]. SVM has been widely applied as a classification tool to address biological questions such as gene function prediction [4], protein homolog identification [5], and disease diagnosis [6]. For example, previous studies used SVM and gene expression data for gene function classification [7] and cancer tissue sample classification [8]. Such studies used a single type of experimental data to conduct the analyses. Recently, Zhu et al. developed a network-based SVM approach where they combined prior knowledge with microarray data to improve the predictive performance for cancer tissue diagnostics [9]. In another study, SVM-based predictions were applied to infer gene function by concatenating normalized features from diverse datasets [10]. Hence, there is a trend of combining heterogeneous data-types to improve classification where the SVM approach is the computational method of choice. Here we attempted to use this approach to tackle the task of predicting MSMGs utilizing two types of high-throughput data by combining several independent studies.

We hypothesized that we can utilize data from mESCs-related mRNA microarrays profiling and genome-wide transcription factor binding profiling (ChIP-seq) applied to characterize mESCs to classify genes important for ES cell self-renewal and pluripotency (MSMGs). We believe that within these datasets there are subtle patterns from which a gene's functional characteristic, in regards to the self-renewal and pluripotency involvement, Yes or No question, can be inferred. We employed an SVM-based approach to construct a classifier that can be used to predict the class membership as being MSMG or not-MSMG for genes by combining genome-wide mRNA expression profiling data and ChIP-seq data. The accuracy and generality of the classifier are evaluated using the leave-one-out-cross-validation (LOOCV) approach. We also compared the SVM classifier with other machine learning classification methods, including linear discriminant classifier, decision trees, and artificial neural networks. Furthermore, we tested the ability of the SVM classifiers to predict the class membership of positive and negative lists of genes resulting from two genome-wide RNAi screen studies to demonstrate how such classification approach can be useful for helping in prioritizing hits from such screens.

## Results

### Learning from heterogeneous data types

We extracted 91 features/attributes from mRNA gene expression and ChIP-seq experiments for each gene (vector) from mESCs-related studies (detailed description is provided in the Methods section). 79 features/attributes were created from mRNA expression microarray profiling data extracted from the Gene Expression Omnibus (GEO) database [12] references to the files are provided in the methods and Additional files 1, 2 and 3. In addition to the 79 features/attributes created from mRNA expression microarray data, we produced 12 features/attributes from ChIP-seq studies [13]. All 12 ChIP-seq experiments we used profile the global genome-wide binding of transcription factors known to be important for maintaining self-renewal and pluripotency [14]. We implemented two types of preprocessing approaches for generating features/attributes from the ChIP-seq datasets: With the first approach, we converted the results from the ChIP-seq experiments into Boolean values where zero represents absence and one represents presence of binding sites in proximity to a gene detected as a peak in a ChIP-seq experiment. The second approach for creating features from the ChIP-seq data was to compute a continuous binding value calculated as a weighted sum of intensities of all of the peaks of the transcription factor weighted by the distance between the peak and the transcription start site (TSS) [15]:

(1) $$a_{ij}=\sum_{k}g_{k}e^{-d_{k}/d_{0}}$$

Where *a_ij _*is the binding value of the transcription factor j on gene i, *g_k _*is the intensity of the k^th ^binding peak of transcription factor j, d_k _is the distance between the TSS of gene i and the k^th ^binding peak, and d_0 _is a constant. *a_ij _*is then log-transformed and quantile-normalized. This method was previously introduced by Ouyang et al. [15]. Altogether, the features created from the ChIP-seq datasets are either 12 binary-valued vectors or 12 continuous-valued vectors consequently named *ChIP-binary *and *ChIP-continuous *in the charts and tables.

In order to train any supervised machine-learning classifier it is required to have a gold standard training set of classified examples. In our case, these are genes that are known to be either MSMG or not-MSMG. For this we obtained an expert collection set of genes labeled as MSMG or not-MSMG (Table 1). This classification of genes/proteins was mainly followed from a study that designed a customize microarray for mESCs [16]. In addition, we used manual expert curation process which included the construction of a literature-based self-renewal regulatory network in mESCs from low throughput studies [17]. In all, we obtained 46 genes as positive examples, classified as MSMG, and 70 genes as negative examples (Table 1). The training sample for positive genes is relatively small since we discarded controversial candidates.

**Table 1:** Training set gene list

MSMGs	Non-MSMGs
Bmp4, Cdyl, Cdyl2, Dmrt1, Dppa4,	Afp, Arid3a, Arid3b, Ascl1, Ascl2,
Dppa5a, Esrrb, Etv4, Etv5, Fgf4, Foxd3,	Bat1a, Bmp2, Bmp5, Bmper, Ccnd2,
Foxh1, Gbx2, Grhl2, Jarid2, Klf2, Klf5,	Cdh2, Cebpa, Cited1, Dach1, Dlx1,
Lefty2, Lin28, Mkrn1, Mycn, Nanog,	Dlx4, Dlx6, Ednra, En1, Eomes, Ets2,
Nodal, Nr0b1, Nr5a2, Phc1, Phf17,	Eya2, Fgf5, Foxb1, Gata1, Gata3,
Pou4f2, Pou5f1, Rif1, Sall1, Sall4, Sgk1,	Gata4, Gata5, Gata6, Gfap, Gli3, Gsc,
Slc27a2, Socs3, Sox2, Spp1, Tcf15,	Hand1, Hand2, Insm1, Isl1, Lbx1,
Tcfap2c, Tcfcp2l1, Tcl1, Tle4, Trp53,	Lhx2, Lhx5, Lmx1a, Mbd2, Meis1,
Utf1, Zfp296, Zfp42	Mixl1, Myf5, Neurog1, Nfia, Npas3,
	Nr2f1, Nr2f2, Nrp1, Nrp2, Olig3, Otp,
	Otx1, Pax3, Pdx1, Peg3, Phox2b,
	Prl3d1, Prox1, Rybp, Shh, Sox1,
	Sox18, Sox3, Sox5, Sox9, Stra13, Syp,
	Tcf4

List of genes used as training set include 46 positive examples labelled as MSMG class and 70 negative examples labelled as non-MSMG class. These genes are derived from expert curation.

**Figure 1:**
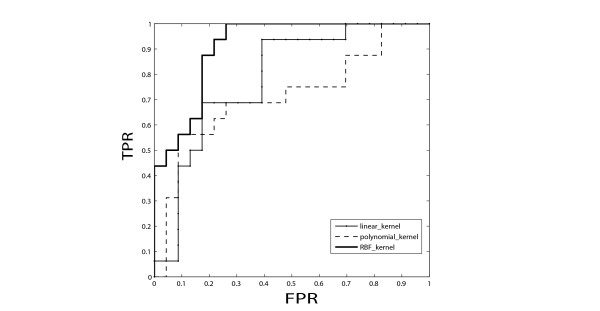
**ROC curves**. Representative ROC curves for three kernel-based SVM classifiers generated using the threefold cross-validation with the mRNA expression microarray dataset for training only. The ROC curves were generated by varying the decision threshold of each SVM classifier. The average AUC for the linear kernel, polynomial kernel and RBF kernel are 0.89, 0.85, and 0.95, respectively. ROC: receiver operating characteristic; TPR: true positive rate; FPR: false positive rate; AUC: area under the curve.

In the first set of computational experiments we tested different versions of SVM classifiers and combinations of training data types to determine which kernel function and which data type or combination of data-types performs best. Table 2 summarizes such evaluations using the LOOCV. In general, the performance of SVM on combined data types, microarray data and ChIP-seq data, appears to perform better than SVM trained on an individual data type. Additionally, the LOOCV results show that the SVM classifier with the radial basis function (RBF) kernel function appears to perform slightly better than classifiers with linear or polynomial kernel functions. The RBF function kernel used is a Gaussian radial basis function with a gamma variable that ranges between 0.1-10 as determined through the outer loop of the LOOCV selected based on the highest accuracy. In addition to the LOOCV, we also employed a three-fold cross-validation in order to plot a receiver operating characteristic (ROC) curve to compute an area under the curve (AUC) score (Figure 1).

**Table 2:** Performance of SVM classifiers

Datatype_kernel	TP	FP	TN	FN	TPR	FPR	Accuracy
micro_linear	42	17	53	4	0.91	0.24	0.82

micro_poly	39	24	46	7	0.85	0.34	0.73

**micro_RBF**	37	3	67	9	0.80	0.04	**0.90**

chip_binary_linear	35	10	60	11	0.78	0.13	0.84

chip_binary_poly	36	5	65	10	0.78	0.07	0.87

**chip_binary_RBF**	39	8	62	7	0.85	0.11	**0.87**

chip_contin_linear	38	7	63	8	0.83	0.10	0.87

chip_contin_poly	36	8	62	10	0.78	0.11	0.84

**chip_contin_RBF**	39	5	65	7	0.85	0.07	**0.90**

weight_binary_linear	39	9	61	7	0.85	0.13	0.86

weight_binary_poly	37	5	65	9	0.80	0.07	0.88

weight_binary_RBF	40	4	66	6	0.87	0.06	0.91

weight_contin_linear	41	9	61	5	0.89	0.13	0.88

weight_contin_poly	37	8	62	9	0.80	0.11	0.85

**weight_contin_RBF**	42	5	65	4	0.91	0.07	**0.92**

simple_binary_linear	39	9	61	7	0.85	0.13	0.86

simple_binary_poly	37	3	67	9	0.80	0.04	0.90

**simple_binary_RBF**	42	3	67	4	0.91	0.04	**0.94**

simple_contin_linear	41	9	61	5	0.89	0.13	0.88

simple_contin_poly	43	17	53	3	0.93	0.24	0.83

**simple_contin_RBF**	41	3	67	5	0.89	0.04	**0.93**

Comparison of performance of several kernel functions used for SVM learning applied on single and heterogeneous data types (mRNA expression and ChIP-seq). The best performer for each category is bold-highlighted. Kernel functions include: linear kernel, polynomial kernel (poly) and Gaussian radial basis kernel (RBF) (see methods). Datasets include: micro-mRNA expression microarrays; chip_binary-ChIP-seq data with pre-processing into binary feature values; chip_contin-ChIP-seq data with pre-processing into continuous feature values. Performance of two data integration strategies: "weight"- weighted kernel matrices; "simple"- one kernel matrix by concatenation of the two data types (see methods). As an example, "simple_binary_poly" means the approach of concatenating microarray and binary ChIP-seq data and training using an SVM with a polynomial kernel function.

An alternative approach to using different individual SVM kernel functions with all the features/attributes is to combine two or more SVM kernel functions for optimizing performance. In a prior similar study it was shown that using different kernels on heterogeneous datasets works better for gene function classification [5]. We did not observe much advantage of implementing weighted combinations of kernels applied to each data type separately. The reason may be the different data types used, ChIP-seq and mRNA expression microarrays data in our study versus phylogenetic and mRNA expression microarrays data in the other study. ChIP-seq and mRNA expression microarrays data is intuitively more correlated [15].

Next we asked which features/attributes/studies contribute the most for successful classification of MSMG genes. For this purpose we implemented a feature selection and ranking algorithm. We applied the SVM Recursive Feature Elimination (RFE) algorithm [18] to rank all features for evaluating their discriminatory capabilities. The top 20 discriminatory features from the RBF-SVM and Poly-SVM classifiers are listed in Additional file 4 which includes both data types (microarray and ChIP-seq). Applying SVM-RFE on both classifiers (RBF-SVM and Poly-SVM) we identified many overlapping features (Fisher's exact, p-value < 0.01).

In summary, we show that the SVM-based classification can be successfully applied for discriminating between MSMG and non-MSMG, whereas combining heterogeneous data types improves learning.

### SVM outperforms other classification methods

In the second set of computational experiments we compared the performance of the SVM classifiers to the following four other types of machine learning classification methods: Linear Discriminant Analysis (LDA) [19], Decision Trees (DT) [20], Artificial Neural Network (ANN) [21], and a simple classifier we created by comparing genes expressed in mESCs vs. genes expressed in embryonic bodies (EB). LDA uses training data to estimate the parameters of discriminant functions which determine boundaries in predictor space between various classes. Alternatively, DT offer a nonparametric model generating a classification tree where each branched node is split based on the values of features of gene vectors computed using information theory. ANN contain an input layer that takes in the feature values, a hidden layer made of nodes connected to the input layer with weighted links that can be adjusted, and an output layer consisting of the resultant classification. In addition, to rule out the possibility that the SVM and other classifiers simply detect mESC-specific genes, we also compared our methods to a simple classifier which predicts MSMGs based only on the gene expression fold change between mESCs and EB cells.

**Figure 2:**
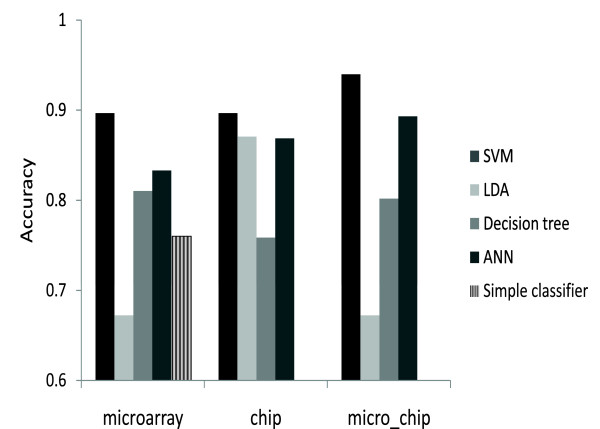
**Classification performance of different types of classifiers**. The performance of the best SVM in each category is compared to three other standard machine learning methods: LDA (Linear Discriminant Analysis), Decision Tree, and ANN (Artificial Neural Networks) and a simple fold-change-based predictor. Performance of machine learning methods is evaluated and accuracy is measured using LOOCV. Labelling of panels is as follows, "microarray": using genome-wide mRNA microarray profiling data; "chip": using genome-wide ChIP-seq of transcription factors data; "micro-chip": using both microarray and ChIP-seq. The fold-change-based predictor results are only under the "microarray" panel since it uses only microarray data.

Figure 2 summarizes the results of comparison between the different classification methods. In general, the results show that the SVM classifier outperforms the other methods. In all cases the best trained SVM either outperforms or is comparable to the other methods. However, these are not conclusive results since we haven't attempted to optimize parameter settings for the other classification methods. The average prediction accuracy of the simple classifier is 0.76 indicating that comparing the fold change for genes expressed in mESCs to EB cells is predictive by itself; however, this approach does not perform as well as any of the other classifiers.

### Prioritizing candidate genes from genome-wide RNAi screens

**Figure 3:**
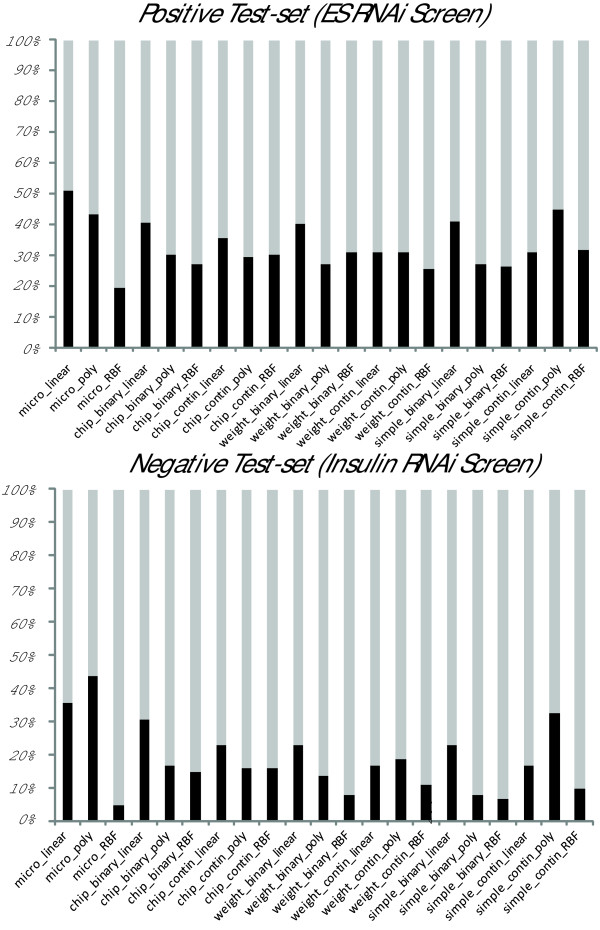
**SVM classifiers to prioritize candidate genes from genome-wide RNAi screens**. Application of SVM classifiers to predict "stemness" genes applied on test sets of two independent genome-wide RNAi screens that identified candidate genes functional for self-renewal and insulin cell signalling. The black bars show the percentage of predicted MSMGs among the total genes from (a) positive test set (functional in self-renewal); and (b) negative test set (functional in insulin signalling).

The third set of computational experiments further test the generality of the SVM-based MSMG prediction classifier. Here we aim to assess whether genome-wide experimental characterization of genes, such as those data produced by mRNA expression profiling and genome-wide transcription factor binding profiling, can truly confer functional description (i.e. self-renewal and pluripotency membership). With this question in mind, we choose two independent studies to generate two test-sets of genes as positive and negative examples. The positive example test-set comes from a study that identified candidate genes functional in maintaining mESCs self-renewal using a genome-wide RNAi screen [22]. Whereas the negative test-set are genes identified as being important for the insulin signaling pathway, also identified from another genome-wide RNAi screen [23]. The insulin pathway related screen is considered as irrelevant to our MSMG definition and MSMG prediction task. However, we cannot rule out the possibility that some genes from the negative example test-set are also involved in stem-cell self-renewal and pluripotency regulation. The ratio (percentage) of predicted MSMG genes from the positive and negative test-set samples can be viewed as "signal-to-noise" ratio (Table 3). Overall, regardless of the data type used, whether we use microarray data alone or integrated data from microarrays and ChIP-seq, the number of genes predicted to be MSMG from the positive test set is significantly higher than from the negative test set (p-value ≈ 5.34 × 10^-12^, two-tail t-test) (Figure 3). Additionally, there is a high correlation (r = 0.89, Spearman's rank correlation) between the prediction accuracy from the LOOCV evaluation of SVMs and the signal-to-noise ratio generated from SVM predictions on the independent RNAi datasets. In other words, the prediction capacity of the SVM for future samples can be well estimated from its performance on our test-set examples using the LOOCV method. Hence, the SVM classifier is capable of discriminating between relevant RNAi screens hits and not relevant hits from another RNAi screen.

**Table 3:** Evaluation of RNAi screens as a test set

Datatype_kernel	Signal-to-noise ratio
micro_linear	1.44

micro_poly	1.00

**micro_RBF**	**3.91**

chip_binary_linear	1.36

chip_binary_poly	1.84

**chip_binary_RBF**	**1.88**

chip_contin_linear	1.57

chip_contin_poly	1.86

**chip_contin_RBF**	**1.91**

weight_binary_linear	1.80

weight_binary_poly	1.96

**weight_binary_RBF**	**3.82**

weight_contin_linear	1.84

weight_contin_poly	1.65

**weight_contin_RBF**	**2.35**

simple_binary_linear	1.80

simple_binary_poly	3.43

**simple_binary_RBF**	**3.80**

simple_contin_linear	1.84

simple_contin_poly	1.38

**simple_contin_RBF**	**3.21**

Ranked methods based on signal-to-noise ratio performance of predicting the percentage of genes as positive from the positive test set (self-renewal screen) and as positive from the negative test set (insulin-pathway screen).

Given the labor-intensive effort and cost of identifying candidate genes from large-scale RNAi screens, the classifiers developed here may help in further prioritizing hits for functional experimental verification. Genome-wide RNAi screens are considered noisy, containing high degree of false positives, where slightly different experimental protocols and statistical analyses can yield different results. As an example, recently Ding's group [24] demonstrated how a genome-wide RNAi screen approach was used to identify novel regulators of embryonic stem cell maintenance. Their results reveal a small overlap with the study we used here as a test-set [22]; 11 out of 209 candidate genes from the RNAi screen implemented by Ding's group overlaps with the study we used here in which 148 candidates were reported. Taken this into consideration, future work should continually test and train classifiers by using diverse data types to build more robust predictions of MSMGs.

### Misclassified genes are also of interest

Interestingly, across various trained SVM classifiers some genes from the stem-cell RNAi screen universally resulted as being false positives regardless of the choice of data type or SVM kernel function used. All the results of the predictions made with various kinds of SVM classifiers are available as Additional file 5. Hence, it is possible that these negative examples are potentially misclassified and are putative MSMG genes, functional in self-renewal and pluripotency maintenance. For example, the gene *Rybp *(RING1 and YY1 binding protein) is labeled as a negative example, but consistently predicted as MSMG. Through careful examination of the literature we found that *Rybp *plays a role during early embryonic development [25]. Similarly, misclassified genes are also found in the genome-wide insulin signaling pathway RNAi screen. Specifically, we found several candidate genes that are always predicted as MSMG, for example, *Pim3 *and *Tnk2*. It was shown that self-renewal of mESCs is supported by *Pim1 *and *Pim3 *[26], whereas *Tnk2 *was reported to stimulate breast cancer development in humans [27]. Considering the relation between stem cells self-renewal maintenance and cancer cells development, *Tnk2 *also appears to be a promising candidate for qualifying as a bona-fide MSMG. We emphasize that the misclassified genes, initially identified as critical for insulin signaling pathway, do not appear in our training set and therefore never seen by the SVM classifier before. Nevertheless, the classifier consistently predicted them as MSMGs.

## Discussion

In this study we demonstrate the ability of SVM classifiers to predict MSMG membership. The results confirm that SVM is a fine choice for this type of classification task for this type of data. Since genome-wide RNAi screens used for discovering functional genes in stem-cell self-renewal and pluripotency maintenance produce candidate lists that are inherently noisy, the SVM-based classifier can be applied to prioritize experimental choices when facing with a large list of candidate genes to verify and further functionally characterize. SVM has the advantage of being flexible for handling different data types as features in an input vector. This facilitates combining various data sources complementing each other which in general we show can increase accuracy. In our study, we only used pre-translational data. In other words, genes can only be differentiated from other genes at the mRNA and protein/DNA interaction levels. This means that post-translational properties cannot be correctly learned. Fortunately, with the growing availability of high-throughput data at the proteome level, i.e. phosphoproteomics profiling of embryonic stem cells [28,29], classification methods such as the one developed here have the potential to increase their prediction accuracy by combining such datasets.

Our computational experiments demonstrate that in general the SVM classifiers benefit from incorporating heterogeneous data. However, learning from various data types was not beneficial for the LDA and Decision Trees classifiers (Figure 2). The decrease in performance for LDA and Decision Trees might be due to sensitivity to features that do not provide substantial contribution to the classification. In addition, we did not implement a search for optimal parameter settings and feature selections for those classifiers. This would probably allow better performance and assessment of the different types of classifiers we tested.

When training a classifier to predict gene membership such as MSMG, there is a tradeoff between the size of the training set and the accuracy that can be achieved. In this study we chose to use a relatively small yet more reliable training set to increase our certainty about the true positives and true negatives. Alternatively, we could integrate together, as positive and negative training sets, genes identified from various studies, including both high-throughput and labor-intensive small-scale approaches. It would be interesting to see if such an approach would improve the performance of MSMG classification.

## Conclusions

In summary, our results reveal that SVM classifiers are useful for predicting genes important for self-renewal and pluripotency of mESCs. Such an approach can be useful for prioritizing genes for functional experiments and complement the analyses of high-throughput profiling experimental data in stem cell research.

## Methods

### Data selection and preprocessing

To minimize the variability of different platforms and inconsistency that arise from gene ID mapping, we extract expression profile data collected from only two Affymatrix platforms (GPL339 and GPL340). These studies include time-series differential expression data from perturbed mouse ES and ES-derived cell lines [GEO: GSE3223, GSE3231, GSE2972, GSE4679] [30,31]. The 79 microarray experiments reflect gene expressions in diverse contexts, including expression profiling for mESCs and Embryoid Bodies (EB) (GSE3223), time-course expression profiling for V6.4 ES cell differentiation (GSE3231) and for R1 ES cell differentiation (GSE2972), time-course expression profiling for RA-induced ES cell differentiation, Esrrb-knockdown-ES cells, Nanog-knockdown-ES cells, Oct4-knockdown-ES cells, Sox2-knockdown-ES cells, Tbx3-knockdown-ES cells as well as Tcl1-knockdown-ES cells (GSE4679). More specifically, GSE3223 reports 12 arrays comparing gene expression in ES cells (J1-ES) and EB cells (J1-EB) each in triplicates using two arrays (MOE430A and MOE430B). Therefore, we extracted two features from this study: the first feature is for each gene averaged expression in J1-ES and the second feature for each gene's average in J1-EB. GSE3231 and GSE2972 have 66 arrays: these two datasets profiled 11-time-points gene expression of V6.5 and R1 ES cells under undirected differentiation, with each time point measured as triplicate and each repeat using two arrays (MOE430A and MOE430B) to cover the whole genome. Therefore, we extracted 11 features from each dataset (V6.5 and R1). GSE4679 holds 140 arrays including: (1) Seven time-points (including day 0) of gene expression of differentiating ES cells under RA-induced differentiation culture conditions, with each time point having one sample on two arrays (MOE430A and MOE430B). We extracted seven features from this dataset. Each feature is made of gene expression at a specific time-point. (2) Eight time-points (including day 0) of gene expression from ES cells where each sample is from two arrays (MOE430A and MOE430B) and has a knock-down vector for: Esrrb shRNAi, Nanog shRNAi, Oct4 shRNAi, Mm343880 shRNAi, Tbx3 shRNAi, Tcl1 shRNAi, Sox2 shRNAi, and an empty vector. We extracted eight time-point features for each of the following six samples: Esrrb, Nanog, Oct4, Tbx3, Tcl1, and Sox2, for a total of 48 features. We did not include the empty vector and the Mm343880 knockdown. More details are provided in Additional files 1, 2 and 3. All experiments were done in the mouse. Expression values were converted to features for each gene (as a vector, X). Expression values were log-transformed and scaled as follows:

(2) $$X_{i}=\frac{\log2\left(E_{i}\right)}{\sqrt{\sum_{i=1}^{79}{\left(\log2\left(E_{i}\right)\right)}^{2}}}$$

This method for preprocessing microarray data for SVM training was borrowed from Brown et al. [8].

### Weighted kernel functions

The SVM classifiers we implemented to map the data from the input space to a high-dimensional space in which classification can be performed by locating data points with respect to a hyperplane that separates binary classes. The feature space can be adjusted by selecting a kernel function, which is used to transform the data for optimization of the classification [4,19]. In this study we utilized three common kernel functions and compared their prediction accuracy:

(1) Linear kernel:

(3) K(X,Y)=X·Y

(2) Polynomial kernel:

(4) $$K\left(X,Y\right)={\left(X\cdot Y+1\right)}^{d}\text{, here we used degree d equals to 3}$$

(3) Gaussian radial basis kernel

(5) $$K\left(X,\;Y\right)=e^{\frac{|X-Y{|}^{2}}{2\sigma^{2}}}$$

It is common practice to set σ to be equal to the median of the Euclidean distances from each positive sample vector to its closest negative sample vector [6], we note that such choice was not optimal for this particular application. Therefore, our strategy for determining σ is to sample a range of values (10^-2 ^to 10^1^), using LOOCV, to select the best σ value that maximizes the prediction accuracy.

### Classifier training

We integrated the two data types, mRNA expression and ChIP-seq, simply by concatenation. We then trained the SVM classifier using one of three kernel functions. Alternatively, motivated by the hypothesis that the classification would be better for treating each data type separately, we employed a strategy of integrating two kernel functions each applied to one of the two different data types. The weights for each classifier are determined by an F1 score.

(6) F1 = 2TP/(2TP+FP+FN)

F1 score is a measure of accuracy that takes into accounts both precision and recall.

(7) $$precision\;=\;\frac{TP}{TP+FP}$$

(8) $$recall\;=\frac{TP}{TP+FN}$$

As a result, we can obtain a classifier that is made of two weighted kernels.

(9) $$K\left(X,\;Y\right)\;=\;F1_{K_{m}}\times K_{m}+F1_{K_{c}}\times K_{c}$$

*K_m _*and *K_c _*represent the kernel matrix measured from the microarray data and the ChIP-seq data, respectively. Hence, the more accurate, the more weight each data type in the kernel matrix would be. The motivation for combining kernel matrices by their weights is that each kernel matrix (from single data type) should exert their effects on the final training of the SVM according to their performance. We named these strategies "simple" and "weighted" in the figures and tables.

### Comparison to other classification methods

Analyses for LDA, Decision Trees and ANN were performed with the default settings using the Statistics Toolbox in MATLAB, Natick, MA. For ANN, we used the Neural Network Toolbox in MATLAB implementing back-propagation to learn a two-layered-feed-forward network with five neurons in the hidden layer. To increase the reliability of the ANN results, we trained 30 ANNs and the final result is computed as the average accuracy. The simple fold-change-based predictor/classifier classifies genes as MSMG if the ESC-to-EB gene expression ratio is more than one. Such ratio was extracted from studies that compared gene expression in mESCs and EBs (GEO accessions: GSE3223, GSE10518). The accuracy of this simple predictor based on these two independent datasets is 0.73 and 0.79, respectively.

### Leave-one-out cross-validation (LOOCV)

The performance of SVM classifiers and other machine learning classification methods is evaluated by LOOCV. Each classifier is trained on n-1 of the total n training samples and tested on the one left out. This step iterates n times to calculate the average performance of the trained classifier as an estimation of prediction error for unseen samples. We measured the accuracy to assess the learning performance:

(10) $$\text{Accuracy }=\frac{TP+TN}{TP+TN+FP+FN}$$

### ROC curves

We also evaluated the performance of various SVM classifiers by measuring the average area under the curve (AUC) using a receiver operating characteristic curve (ROC). Each time we left one fold of training samples out as a testing set and trained the SVM on the other two folds. This step is iterated three times and the average performance can be calibrated as AUC. The ROC curves were generated by varying the decision threshold of each SVM classifier.

### Application of the SVM-based classifier to indentify and classify unseen MSMGs

To evaluate the SVM classifier, we chose two independent sets of genes from genome-wide RNAi screens. One screen identified genes important for stem cell self-renewal and pluripotency, whereas the other screen identified genes important to insulin signaling. We were able to match the IDs of 126 genes from the screen that identified stem cell self-renewal out of 148 genes identified in the study [22]. These genes were used as the positive example test set. 101 genes out of the 126 genes identified as insulin signaling pathway members from the second study were ID matched [23] and used as the negative test set. SVM classifiers trained on our original training set of 46 positive and 70 negative examples were tested for their ability to classify genes from these two independent sets. The ratio of percentage of predicted MSMGs from the positive and negative test samples can be viewed as a signal-to-noise ratio:

(11) $$ratio=\frac{\text{Predicted MSMGs in positive set}\%}{\text{Predicted MSMGs in negative set}\%}$$

## Authors' contributions

H.X. and A.M. designed the project. H.X performed the data processing and analysis. H.X, I.R.L and A.M participated in several discussions. H.X and A.M wrote the paper. All authors read and approved the final manuscript.

## Supplementary Material

Additional file 1 **Feature description and reference**. This file contains a table listing all features/attributes with a description and the GEO accession numbers. Click here for file

Additional file 2 **Processed microarray and binary ChIP-seq features for all training and RNAi test samples**. This file contains the normalized microarray features (1 to 79) and binary ChIP-seq features (Feature 80 to 91) for all training and RNAi test samples. Click here for file

Additional file 3 **Processed microarray and continuous ChIP-seq features for all training and RNAi test samples**. This file contains the normalized microarray features (Feature 1 to 79) and continuous ChIP-seq features (Feature 80 to 91) for all training and RNAi test samples. Click here for file

Additional file 4 **Top 20 features**. This file contains two lists that are the top 20 features using the weight magnitudes in a RBF-SVM classifier and a Poly-SVM classifier as criteria for inclusion in the list. This means that these features are most useful for separating the data into the correct classes. Click here for file

Additional file 5 **Results of gene membership predictions of SVM classifiers**. This file contains the classifications results across all SVM classifiers for all training samples and RNAi test samples. The column labels correspond to the same labels as in Table 2 and 3. Click here for file

## References

[B1] Ivanova NB Dimos JT Schaniel C Hackney JA Moore KA Lemischka IR A stem cell molecular signature Science 2002 298 601 604 10.1126/science.1073823 12228721

[B2] Ramalho-Santos M Yoon S Matsuzaki Y Mulligan RC Melton DA "Stemness": transcriptional profiling of embryonic and adult stem cells Science 2002 298 597 600 10.1126/science.1072530 12228720

[B3] Bhattacharya B Miura T Brandenberger R Mejido J Luo Y Yang AX Joshi BH Ginis I Thies RS Amit M Lyons I Condie BG Itskovitz-Eldor J Rao MS Puri RK Gene expression in human embryonic stem cell lines: unique molecular signature Blood 2004 103 2956 2964 10.1182/blood-2003-09-3314 15070671

[B4] Pavlidis P Weston J Cai J Noble WS Learning gene functional classifications from multiple data types J Comput Biol 2002 9 401 411 10.1089/10665270252935539 12015889

[B5] Jaakkola T Diekhans M Haussler D Using the Fisher kernel method to detect remote protein homologies Proc Int Conf Intell Syst Mol Biol 1999 149 158 10786297

[B6] Moler EJ Chow ML Mian IS Analysis of molecular profile data using generative and discriminative methods Physiol Genomics 2000 4 109 126 11120872 10.1152/physiolgenomics.2000.4.2.109

[B7] Brown MP Grundy WN Lin D Cristianini N Sugnet CW Furey TS Ares M Haussler D Knowledge-based analysis of microarray gene expression data by using support vector machines Proc Natl Acad Sci USA 2000 97 262 267 10.1073/pnas.97.1.262 10618406 PMC26651

[B8] Furey TS Cristianini N Duffy N Bednarski DW Schummer M Haussler D Support vector machine classification and validation of cancer tissue samples using microarray expression data Bioinformatics 2000 16 906 914 10.1093/bioinformatics/16.10.906 11120680

[B9] Zhu Y Shen X Pan W Network-based support vector machine for classification of microarray samples BMC Bioinformatics 2009 10 Suppl 1 S21. 19208121 10.1186/1471-2105-10-S1-S21 PMC2648796

[B10] Guan Y Myers CL Hess DC Barutcuoglu Z Caudy AA Troyanskaya OG Predicting gene function in a hierarchical context with an ensemble of classifiers Genome Biol 2008 9 Suppl 1 S3. 10.1186/gb-2008-9-s1-s3 18613947 PMC2447537

[B11] Vapnik V Statistical Learning Theory 1998 New York: Wiley

[B12] Barrett T Troup DB Wilhite SE Ledoux P Rudnev D Evangelista C Kim IF Soboleva A Tomashevsky M Edgar R NCBI GEO: mining tens of millions of expression profiles--database and tools update Nucleic Acids Res 2007 35 D760 765 10.1093/nar/gkl887 17099226 PMC1669752

[B13] Chen X Xu H Yuan P Fang F Huss M Vega VB Wong E Orlov YL Zhang W Jiang J Loh YH Yeo HC Yeo ZX Narang V Govindarajan KR Leong B Shahab A Ruan Y Bourque G Sung WK Clarke ND Wei CL Ng HH Integration of external signaling pathways with the core transcriptional network in embryonic stem cells Cell 2008 133 1106 1117 10.1016/j.cell.2008.04.043 18555785

[B14] Nishiyama A Xin L Sharov AA Thomas M Mowrer G Meyers E Piao Y Mehta S Yee S Nakatake Y Stagg C Sharova L Correa-Cerro LS Bassey U Hoang H Kim E Tapnio R Qian Y Dudekula D Zalzman M Li M Falco G Yang HT Lee SL Monti M Stanghellini I Islam MN Nagaraja R Goldberg I Wang W Uncovering early response of gene regulatory networks in ESCs by systematic induction of transcription factors Cell Stem Cell 2009 5 420 433 10.1016/j.stem.2009.07.012 19796622 PMC2770715

[B15] Ouyang Z Zhou Q Wong WH ChIP-Seq of transcription factors predicts absolute and differential gene expression in embryonic stem cells Proc Natl Acad Sci USA 2009 106 21521 6 10.1073/pnas.0904863106 19995984 PMC2789751

[B16] Jiang J Chan YS Loh YH Cai J Tong GQ Lim CA Robson P Zhong S Ng A core Klf circuitry regulates self-renewal of embryonic stem cells Nat Cell Biol 2008 10 353 360 10.1038/ncb1698 18264089

[B17] Xu H Schaniel C Lemischka IR Ma'ayan A Toward a complete in silico, multi-layered embryonic stem cell regulatory network Wiley Interdiscip Rev Syst Biol Med 2010 2 708 733 20890967 10.1002/wsbm.93 PMC2951283

[B18] Guyon I Weston J Barnhill S Vapnik V Gene selection for cancer classification using support vector machines Machine Learning 2002 46 389 422 10.1023/A:1012487302797

[B19] Cristianini N SHawe-Taylor J An introduciton to Support Vector Machines 2000 Cambridge: Cambridge University Press

[B20] Bishop CM Pattern Recognition and Machine Learning 2006 New York: Springer Science + Business Media

[B21] Quinlan JR Induction of Decision Trees Machine Learning 1986 1 81 106

[B22] Hu G Kim J Xu Q Leng Y Orkin SH Elledge SJ A genome-wide RNAi screen identifies a new transcriptional module required for self-renewal Genes Dev 2009 23 837 848 10.1101/gad.1769609 19339689 PMC2666338

[B23] Tu Z Argmann C Wong KK Mitnaul LJ Edwards S Sach IC Zhu J Schadt EE Integrating siRNA and protein-protein interaction data to identify an expanded insulin signaling network Genome Res 2009 19 1057 1067 10.1101/gr.087890.108 19261841 PMC2694478

[B24] Ding L Paszkowski-Rogacz M Nitzsche A Slabicki MM Heninger AK de Vries I Kittler R Junqueira M Shevchenko A Schulz H Hubner N Doss MX Sachinidis A Hescheler J Lacone R Anastassiadis K Stewart AF Pisabarro MT Caldarelli A Poser I Theis M Buchholz F A genome-scale RNAi screen for Oct4 modulators defines a role of the Paf1 complex for embryonic stem cell identity Cell Stem Cell 2009 4 403 415 10.1016/j.stem.2009.03.009 19345177

[B25] Pirity MK Locker J Schreiber-Agus N Rybp/DEDAF is required for early postimplantation and for central nervous system development Mol Cell Biol 2005 25 7193 7202 10.1128/MCB.25.16.7193-7202.2005 16055728 PMC1190233

[B26] Aksoy I Sakabedoyan C Bourillot PY Malashicheva AB Mancip J Knoblauch K Afanassieff M Savatier P Self-renewal of murine embryonic stem cells is supported by the serine/threonine kinases Pim-1 and Pim-3 Stem Cells 2007 25 2996 3004 10.1634/stemcells.2007-0066 17717068

[B27] Howlin J Rosenkvist J Andersson T TNK2 preserves epidermal growth factor receptor expression on the cell surface and enhances migration and invasion of human breast cancer cells Breast Cancer Res 2008 10 R36 10.1186/bcr2087 18435854 PMC2397538

[B28] Brill LM Xiong W Lee KB Ficarro SB Crain A Xu Y Terskikh A Snyder EY Ding S Phosphoproteomic analysis of human embryonic stem cells Cell Stem Cell 2009 5 204 213 10.1016/j.stem.2009.06.002 19664994 PMC2726933

[B29] Van Hoof D Munoz J Braam SR Pinkse MW Linding R Heck AJ Mummery CL Krijgsveld J Phosphorylation dynamics during early differentiation of human embryonic stem cells Cell Stem Cell 2009 5 214 226 10.1016/j.stem.2009.05.021 19664995

[B30] Ivanova N Dobrin R Lu R Kotenko I Levorse J DeCoste C Schafer X Lun Y Lemischka IR Dissecting self-renewal in stem cells with RNA interference Nature 2006 442 533 538 10.1038/nature04915 16767105

[B31] Hailesellasse Sene K Porter CJ Palidwor G Perez-Iratxeta C Muro EM Campbell PA Rudnicki MA Andrade-Navarro MA Gene function in early mouse embryonic stem cell differentiation BMC Genomics 2007 8 85 10.1186/1471-2164-8-85 17394647 PMC1851713

